# Beyond glycan barriers: non-cognate ligands and protein mimicry approaches to elicit broadly neutralizing antibodies for HIV-1

**DOI:** 10.1186/s12929-024-01073-y

**Published:** 2024-08-21

**Authors:** Stephen Ian Walimbwa, Petr Maly, Leona Raskova Kafkova, Milan Raska

**Affiliations:** 1 Department of Immunology, University Hospital Olomouc, Zdravotníků 248/7, 77900 Olomouc, Czech Republic; 2 Laboratory of Ligand Engineering, Institute of Biotechnology of the Czech Academy of Sciences, BIOCEV Research Center, Průmyslová 595, 252 50 Vestec, Czech Republic; 3 Department of Immunology, Faculty of Medicine and Dentistry, Palacky University Olomouc, Hněvotínská 3, 779 00 Olomouc, Czech Republic

**Keywords:** HIV-1 vaccine, Glycans, Broadly neutralizing antibodies, Protein mimicry, Combinatorial protein library, Non-cognate ligands

## Abstract

Human immunodeficiency virus type 1 (HIV-1) vaccine immunogens capable of inducing broadly neutralizing antibodies (bNAbs) remain obscure. HIV-1 evades immune responses through enormous diversity and hides its conserved vulnerable epitopes on the envelope glycoprotein (Env) by displaying an extensive immunodominant glycan shield. In elite HIV-1 viremic controllers, glycan-dependent bNAbs targeting conserved Env epitopes have been isolated and are utilized as vaccine design templates. However, immunological tolerance mechanisms limit the development of these antibodies in the general population. The well characterized bNAbs monoclonal variants frequently exhibit extensive levels of somatic hypermutation, a long third heavy chain complementary determining region, or a short third light chain complementarity determining region, and some exhibit poly-reactivity to autoantigens. This review elaborates on the obstacles to engaging and manipulating the Env glycoprotein as an effective immunogen and describes an alternative reverse vaccinology approach to develop a novel category of bNAb-epitope-derived non-cognate immunogens for HIV-1 vaccine design.

**Graphical Abstract:**

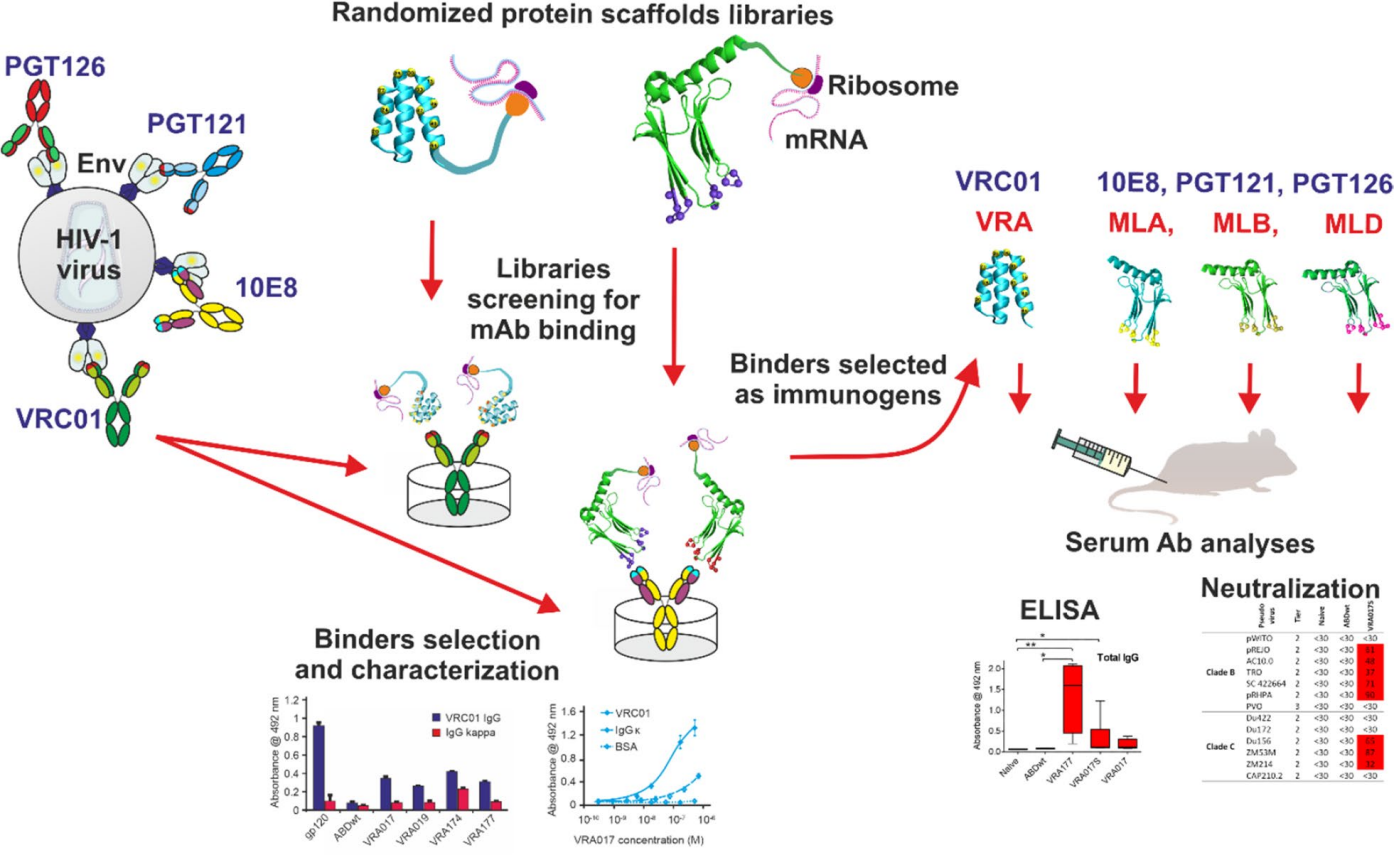

## Introduction

An effective vaccine is urgently needed to prevent the human immunodeficiency virus type 1 (HIV-1) epidemic, a disease transmitted to approximately 1.5 million people each year [1]. Currently, lifelong antiretroviral therapy (ART) combinations are the standard of care for the management of HIV in people living with HIV (PLHIV). However, ART does not cure or eradicate the virus from infected cells. Antiretroviral therapy efficiently suppresses HIV viral replication, delays disease progression, restores T-cell immune responses, and improves the quality of life and the survival rate of PLHIV [2,3]. Unfortunately, transmissions still occur either from untreated people living with HIV or from PLHIV on antiretroviral therapy with insufficient virological suppression.

The HIV-1 infection begins with the entry and integration of the viral genome into target cells, which is initiated by the binding of a trimeric envelope (Env) spike on the HIV-1 surface to CD4 receptors and co-receptors CCR5 or CXCR4 on host cells [4]. The binding of Env gp120 to the CD4 binding site on the CD4 T cells induces conformational changes to the gp41 ectodomain which permits membrane fusion and deposition of viral RNA into the host cell cytosol [4,5].

These events result in robust humoral immune responses with the production of non-neutralizing and neutralizing antibodies against the Env trimer to halt the HIV-1 virus from infecting host cells [6,7]. However, HIV-1 possesses several counteracting and subvert mechanisms to evade the antibody effector functions. First, the virion surface displays a few distantly spaced functional spikes, hindering the effective interaction with and activation of B cells [8]. Additionally, the virus produces non-functional uncleaved precursor Env glycoprotein (gp160) and monomeric gp120 and gp41 which act as decoy epitopes that divert the antibody response to non-protective responses [4]. Second, conserved epitopes on the Env trimer are rendered inaccessible to neutralizing antibody responses by an extensive *N*-glycan shield and conformational masking [8,9]. Third, the Env trimer undergoes rapid mutations that generate enormous sequence variability that prevails over the antibody responses [10–12].

During the acute HIV-1 phase, functional non-neutralizing antibodies (nNAbs) against Env are rapidly developed in the infected host [8]. The nNAbs protect against viral spreading through multiple binding events and the involvement of additional immune effector mechanisms. nNAbs are typically strain-specific and exhibit antiviral activity via phagocytosis of infectious virions, antibody-dependent cellular cytotoxicity (ADCC), antibody-dependent complement deposition (ADCD), antibody-dependent virus inhibition (ADCVI), and sequestration of Fc receptor-bearing infected T cells [7,12,13]. Notably, nNAbs produced against HIV-1 primarily target the immunodominant gp41 ectodomain but cannot prevent productive (*cis*) infection of target immune cells [12].

Generally, in the absence of antiretroviral therapy, PLHIV progresses to AIDS within 8–10 years after infection [14]. In approximately 20% of PLHIV, often referred to as long-term non-progressors (LTNP), disease progression and the development of clinical signs and symptoms are remarkably delayed in the absence of ART [14–17]. Based on viremic control of HIV-1 viral load, LTNP can be distinguished as viremic controllers and viremic non-controllers [14]. HIV-1 elite-controllers (EC) are a unique subset of approximately 0.3% of LTNP viremic controllers who maintain an undetectable viral load and high CD4 T-cell counts in the absence of ART [17,18]. Elite-controllers exhibit potent broadly neutralizing antibodies (bNAbs) against diverse isolates of HIV-1 which prevent the viral infection of target cells [8]. bNAbs predominately target highly conserved epitope clusters on the Env glycoprotein trimer: CD4 binding site on gp120, V3 loop within the glycan shield, V2 apex on gp120, glycosylated silent face on gp120, gp120/gp41 interface, fusion peptide, and the membrane-proximal external region (MPER) on the transmembrane domain of gp41 [8,10,19–21].

Attempts to consistently induce these antibodies to establish either a functional or sterilizing HIV-1 cure through vaccination have been challenging in part due to the unique structural and functional properties of bNAbs. For instance, bNAbs frequently exhibit high levels of somatic hypermutations (SHM) arising from prolonged co-evolution with HIV and are associated with inserts and/or deletions. [22,23] Also, many bNAbs possess exceptionally long heavy chain complementary determining region 3 (CDRH3) or short light chain complementarity determining region 3 (CDRL3), while some exhibit poly-reactivity to host autoantigens. [11,24,25] These immune responses strongly correlate with class I human leukocyte antigen (HLA) polymorphisms on the peptide binding groove which have been associated with the spontaneous control of HIV-1 viral load in elite-controllers. [15] Interestingly, glycan-dependent bNAbs rely on Env trimer architecture and the composition of potential N-glycosylation sites which are considerably conserved among diverse HIV-1 clades and isolates. [26] Alterations in signature glycans which sterically mask conserved epitopes on the Env trimer have been shown to affect viral binding, infectivity, and neutralization [27,28].

Recent advances in glycoimmunology show that the glycan (glycome) repertoire influences innate and adaptive immune responses. [29] Notably, the glycome in tumors, autoimmune disorders, chronic inflammation, and infectious diseases is characterized by aberrant glycosylation. [30,31] Interestingly, several altered glycan structures mediate immune function and cellular processes by influencing interactions at the cell–cell, and cell-pathogen interfaces [32]. For example, in autoimmune disorders (e.g. IgA nephropathy, rheumatoid arthritis, and systemic lupus erythematosus) the lymphocyte surface proteins typically express altered galactose or N-acetylgalactosamine terminated glycans which impact disease pathogenesis and resolution. [29,30] Additionally, the overexpression of β-galactoside binding lectins (galectins 1/3/9) by several tumors (e.g. melanoma, Hodgkin’s lymphoma, pancreatic carcinoma, and neuroblastoma) may facilitate tumor immune evasion and modulate anti-tumour immune responses. [29,30,33] Conversely, aberrant glycans may be utilized as templates for carbohydrate-based vaccines or diagnostic/therapeutic biomarkers in cancer and infectious diseases [32,34,35].

The HIV glycan shield interacts with the host cell glycan-binding proteins (lectins), sialic acid-binding lectins (siglecs), and galectins. These interactions affect cellular processes and immunological responses during HIV infection. [29,31,36] Siglecs (siglec 6/7/9) and galectins (galectin 1/3/9) interactions have been implicated in HIV immune dysfunction through the induction of T/B-cell exhaustion, apoptosis, and NK cell inhibition. [31] Emerging evidence indicates that the aberrant changes to sialic acid, core fucose, and galactose significantly affect the non-neutralizing antibody responses to HIV infection. For example, in elite controllers, antibody Fc-mediated antiviral effector functions (ADCC/ADCVI) are skewed towards agalactosylated, afucosylated, and asialylated glycoforms. [29,31,37] Also, HIV-infected replication-competent T cells exhibit enhanced surface fucosylation which is essential for T-cell receptor binding, activation, and signaling. Structural analyses of isolated bNAbs have elucidated the dependence on signature glycans within the HIV glycan shield to elicit potent heterologous neutralization. [33] In this review, we provide an overview of the glycan-related obstacles to eliciting bNAbs and describe a reverse vaccinology non-cognate ligand strategy (NCLS) using protein scaffold mimicry to develop HIV-1 vaccine immunogens.

### Env variability and glycan shielding

Env-glycans are carbohydrate moieties that are added as post-translational modifications to Env proteins produced by host cells before being displayed on the surface of pathogenic viruses. Glycans on Env are vital for protein folding, replication, infectivity and evading host immune responses. [26,38,39] Glycosylation of HIV-1 Env precursor gp160 occurs in the host cell endoplasmic reticulum. It is further processed in the Golgi, where gp160 is also proteolytically cleaved by a furin-type protease to gp120 and gp41 and eventually trafficked to the cell surface for incorporation as a trimer on budding viral particles. [39,40] The Env glycoprotein is a trimer of non-covalently bound gp41/gp120 heterodimers and the sole glycoprotein on the viral surface coded by the HIV genome [41].

Approximately 50% of the gp120 molecular mass is composed of *N*-linked oligomannose, hybrid, and complex glycans which form a mannose-patch and are wholly or partially recognized epitopes by some bNAbs. [38,39] Typically, 27–33 potential *N*-linked glycosylation sites (PNGS) per gp120 protomer exist across HIV-1 clades, which generates enormous Env genetic diversity and contributes to the Env surface variability. [38] HIV utilizes the cell glycosylation pathway to display *N*-linked glycans, which shield conserved epitopes on the Env from immune recognition. [38,41] In transmitted/founder (T/F) viruses which are responsible for establishing HIV infection after mucosal exposure, distinct epitope patterns with higher levels of mannose, sialylation, and core fucosylation have been reported. [42] The T/F glycosylation profile of Env gp120 frequently differs from chronic infection variants by displaying fewer glycans with higher replication fitness. [38] T/F viruses also have shorter hypervariable loops and contain fewer PNGSs than those from the chronic variants [39,42].

### Structural analysis of Env glycoprotein as a bNAb target

**Fig. 1:**
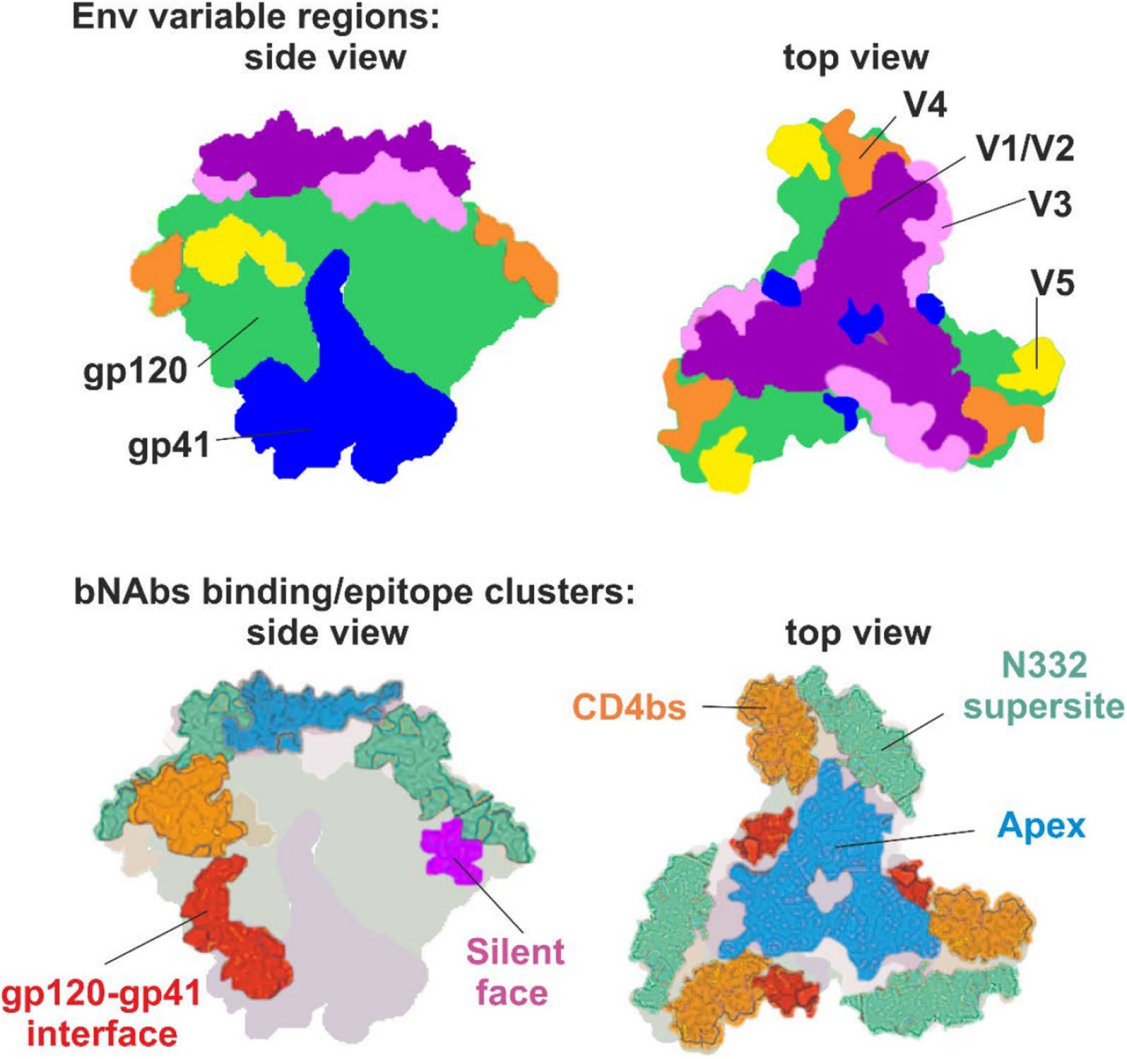
HIV-1 Env trimer structure and the conserved epitopes targeted by broadly neutralizing antibodies (bNAbs). The Env model is based on the cryo-electron microscopy structural model of the BG505 SOSIP.664 trimer available on RSCB PDB under Acc. No. 4ZMJ

The Env crystal structure shows five variable regions (V1-V5) fused with five conserved regions (C1-C5) that are densely glycosylated with N- and O-linked glycans which are prime targets for neutralizing antibodies, and the focus of vaccine development. [23,43,44] Env glycosylation is usually clustered as unprocessed oligomannose *N*-linked glycans, distinctively divergent from typical mammalian cell glycosylation and concentrated at the intrinsic mannose patch on the outer domain of the gp120 subunit and the trimer-associated mannose patch at the trimer apex. [27,40] These glycan clusters are unique targets for bNAbs and are conserved across different HIV-1 group M clades (Fig. 1). [45] In this section, we describe Env trimer epitopes and highlight conserved glycans relevant to HIV-1 vaccine design.

### N332 supersite

This glycan patch is a relatively well conserved region of virus vulnerability on the intrinsic mannose patch with multiple bNAbs heavy and light chain complementary determining region interactions, thus representing a supersite for antibody neutralization. [46,47] N322-dependent bNAbs (e.g. BG18, PGT121, PGT128, PGT130, PGT131, PGT133, PGT136, and PGT137) directly bind to the glycan shield and the GDIR peptide motif at the base of V3 loop inducing conformational changes, which restricts access to this epitope. Notably, 2G12 and PGT135 are GDIR peptide motif independent. [48] The N332 supersite bNAbs possess unique characteristics: first, the molecular orientation of the N332 site is more accessible to various angles of approach which allows diverse binding by glycan-dependent bNAbs to this conserved epitope; second, N332 glycan-directed bNAbs develop after a relatively short period of infection and show lower levels of somatic hypermutation; third, N332-dependent bNAbs exhibit broad interactions with surrounding glycans increasing surface contact with the Env glycoprotein [47,49].

Extensive neutralization breadth has been shown for glycan-specific bNAbs across HIV-1 viral panels, and the absence of vital PNGS on the variable loops may lead to the development of resistance with loss of infectivity. [50,51] During HIV infection, PNGS deletions contribute to immune escape from strain-specific neutralizing antibodies but can also lead to the formation of bNAb epitopes [52,53].

### V1/V2 loop

This domain on gp120 is located at the **apex** of the trimer where it assumes at least three conformational states (β-strand, α-helix, 3_10_ helix) required for Env trimer stabilization, viral entry, neutralization resistance, and integration into the host cell. [54–56] Typically, V1/V2 bNAbs (PG9, PG16, PGT145, and CAP256.09) are elicited by the β-strand conformation and have long CDRH3. [54,57] These bNAbs exhibit 10–20% mutation levels in the variable region of immunoglobulin heavy chain (VH) that target the quaternary epitope on V2 with varying neutralization potency. [58] Interactions between bNAbs and the conserved epitopes on the V1/V2 domain are achieved by the interaction between acidic residues (aspartate and sulphated tyrosine) at the tip of CDRH3 bNAbs and basic lysine-rich residues on the Env spike which provides access to the N160 high mannose glycans, and sialic acid-containing hybrid glycans at positions N156 and N173 (HXB2 numbering). [59] The antibody responses to the V2 apex appear early in natural infection and are developed in several elite-controllers making it a favorable immunogen target albeit difficult to achieve due to the plasticity of Env conformational states during HIV infection. [54,59] In non-human primate, simian-human immunodeficiency viruses (SHIV) challenge experiments, a V2-directed bNAb PGDM1400 demonstrated modest potency and was subsequently transitioned into human clinical trials. Unfortunately, results from clinical trials (NCT03205917) investigating PGDM1400 alone, or in combination with other classes of bNAbs, showed only a temporary reduction in HIV-1 viral load [60].

In the RV144 trial (NCT00223080) where vaccine efficacy was 31.2%, the generation of IgG to V1/V2 loops was associated with a reduction in the risk of acquiring HIV. [55,61] Analysis of the monomeric gp120 immunogens from the RV144 trial also revealed that signature glycans (N135, N141, N156, and N160) on this epitope are vital for inducing antibody responses. [62] Modifications to the heterologous prime-boost approach utilized in the RV144 have demonstrated that heterologous DNA proteins induce sustained humoral responses against V1/V2 loop with broadly binding functional antibodies confirming the durability of this epitope for vaccine development. However, subsequent clinical trials (HVTN705 (NCT03060629) and HVTN702 (NCT02968849)) failed to show similar vaccine efficacy [61].

### V3 loop

The V3 loop is obscured by high-mannose and complex *N*-glycans and is composed of three parts: a crown, stem, and base which contains relatively conserved domains that are vital for HIV-1 entry into target cells via co-receptor CCR5 or CXCR4. [11,63,64] Following CD4-Env binding, conformational changes result in the displacement of the V1/V2 loops exposing the crown on V3, which is vital for CCR5 or CXCR4 co-receptor interaction. [63] Antibodies to the V3 loop crown develop in all PLHIV and are largely non-neutralizing. [64] Therefore, serum from non-human primate prime-boost experiments using multimerized V3 virus-like-particle immunogens elicited weak heterologous neutralization coverage. [65] Conversely, in human clinical trials (ClinicalTrials.gov Identifiers: NCT02960581, NCT02511990), PGT121 and 10-1074 bNAbs exhibited potency and breadth with significant viral load reduction among HIV-infected participants suggesting that these bNAbs can be used as templates in vaccine development [10].

### gp120/gp41 interface containing fusion peptide

The crystal structure of this immune evasive epitope has been visualized when bound to native-like soluble Env trimer SOSIP.664 constructs. [66,67] In the closed/pretriggered conformational state, the gp41 fusion peptide structural core forms a circle of four helices (α6-9) around the N and C termini of gp120. [68] Fluorescence resonance energy transfer studies show that CD4 binding triggers the opening of the Env glycoprotein to induce stable (open) Env trimer conformations. The coreceptor binding allows the fusion peptide transition into an extended six-helix bundle which facilitates viral and target cell membrane fusion. Although bNAbs target the pre-fusion closed metastable Env conformation, antibodies elicited to the open Env conformations are poorly neutralizing [68–70].

Notably, glycan occupancy at potential *N*-linked glycosylation sites within gp120 and the fusion peptide directly affects the stability of the closed Env trimer. [66,68,69] The stability of the Env trimer is also affected by mutations within or near this interface. These Env trimer mutations are associated with loss of HIV infectivity or the emergence of escape mutations at the V1/V2 loop on gp120 and heptad repeat (HR1 and HR2) regions on gp41. [69,71] Interestingly, the variations in glycan composition may directly or allosterically affect paratope binding and the neutralization exhibited by fusion peptide directed bNAbs [72].

Therefore, bNAbs targeting this interface engage and incorporate the conserved complex *N*-linked glycans on gp120 (eg. N88, N611, N234, N276) and gp41 (eg.N241, N637) to exhibit neutralization breadth and potency. [68,69,73] Several glycan-dependent bNAbs (e.g., 8ANC195, PGT151, PGT158, VRC34, 35O22, ACS202, and 3BC315) targeting distinct overlapping epitopes on gp120/gp41 interface located below the CD4 binding site have been isolated in elite controllers. [38,67,74,75] The passive immunization of non-human primates using fusion peptide immunogens elicited bNAbs which effectively protected rhesus macaques against SHIV(BG505) mucosal challenges. [70,76] However, the antibody response to the fusion peptide containing immunogen in a human clinical trial (NCT03783130) showed autologous viral neutralization with no breadth against a BG505 tier-2 strain partly due to glycan masking at the conserved N241 glycan. [70,77] Therefore, the development of immunogens targeting immune responses towards this interface will require ingenuity to decipher glycan heterogeneity and the convergent evolution of viral escape mutants [71,72].

### CD4 binding site

Oligomannose and complex glycans cluster at the distal and proximal positions, respectively, forming a shield that obscures the CD4 binding site (CD4bs). [26] Multiple bNAbs (e.g. b12, VRC01, NIH45-46, VRC-PG04, VRC13, 3BNC117, CH103, N6, N49P7, and IOMA) targeting the CD4bs have been isolated from PLHIV making them attractive candidates for further vaccine development. [11] The CD4bs bNAbs paratopes are derived through either a CDRH3-dominated (loop-dependent antibodies) or VH gene-restricted (CD4-mimic antibodies) ontogeny and disrupt the CD4-Env interactions through VH1-2 or VH1-46 encoded heavy chains. [22,78] VH1-2 heavy chains encode for the VRC01-class antibodies, which are among the most potent and broad CD4bs bNAbs, while VH1-46 encodes for the 8ANC131-class antibodies. [78] VRC01-class antibodies typically have heavy chain residues interacting with gp120, exhibit high levels of SHM required to incorporate the highly conserved N276 glycan which is responsible for thwarting Env-bNAbs interactions, and possess a short five residue CDRL3 which suggests that antibody elicitation through vaccination might be difficult. [11,38,79] Conversely, the IOMA-class bNAbs (e.g., ASC101-3, B24, PCIN7I, PCIN66B, N60P1.1, and N60P25.1) are derived from VH1-2*02 Ig alleles and exhibit fewer SHMs, accommodate the N276 glycan via a short helix in CDRL1 and possess an average-length eight-residue CDRL3, which suggests that this class of bNAbs might be easier to elicit through vaccination [79,80].

In the VRC601 (NCT01950325), HVPTN704 (NCT02716675), and HVTN703 (NCT02568215) clinical trials, VRC01 bNAbs showed strain-specific neutralization and reduction in plasma viremia providing proof-of-concept for developing a therapeutic HIV-1 vaccine [10,81].

### Silent-face

The crystal structure of the silent-face bNAbs paratopes in complex with gp120 has been visualized when bound to native-like BG505 pre-fusion stabilized trimers. [19,21] Cryo-electron microscopy shows that this immunorecessive epitope on gp120 is concealed by highly conserved complex and oligomannose *N*-linked glycans at positions N262, N295, and N448. [9,19,38] These glycans are vital for viral entry and contribute to the generation of immune evasion mechanisms utilized by Env and have also been implicated in emergence of resistance to bNAb neutralization [19,74].

Currently, two rare glycan-dependent bNAbs, VRC-PG05 and SF12 with 27% and 62% neutralization coverage respectively have been isolated targeting the silent face epitope. [19] Several factors contribute to the differences in the neutralization coverage observed. Notably, these two bNAbs have distinct evolutionary pathways arising from somatic hypermutation (SHM) within the VH-gene alleles IgHV (4–59*01 (SF12))) and 3–7*01 (VRC-PG05)) and IgKV (3–20*01 (SF12))) and 4–1*01 (VRC-PG050)). [19,21] Gene analysis of these two bNAbs has shown that the lengths of the amino acid peptides within the heavy and light chain complementary determining region 3 (CDR3) vary between SF12 and VRC-PG05. Notably, to mediate epitope contact and penetrate the glycan shield, SF12 utilizes a heavy chain (CDRH3) of 23 amino acids and a light chain (CDRL3) of 6 amino acids whereas VRC-PG05 uses a CDRH3 of 17 amino acids and CDRL3 of 8 amino acids. [19] These amino acid sequence lengths in the CDR3 regions are similar to the average in the general human antibody repertoire. [82] However, eliciting these bNAbs through vaccination may be challenging because of the unique properties attributed to the extensive SHM and affinity maturation. [19,38] Furthermore, using heavy chains CDRH1 and 2, bNAb SF12 mediates additional contact with amino acid residues on gp120. [19] These additional interactions juxtaposed with gp120 significantly contribute to the observed neutralization by SF12.

Additionally, SF12 can neutralize viruses with the N295 glycan deletion. This contrasts the VRC-PG05 bNAb which requires the presence of the three conserved silent-face masking glycans (N262,N295, and N448) on gp120. [21] Glycans substantially decorate the epitope surfaces targeted by silent-face bNAbs. Therefore, alterations of the Env glycan shield and sequence diversity are unique decoy mechanisms to evade host immune recognition. [38,83] HIV typically escapes antibody neutralization through mutations that lead to viral escape. After treatment with SF12 and VRC-PG05, the viral escape pathways involve modifications to the N448 glycan and glutamic acid residues at position 293 on gp120 [19,21]. Although N448 glycan is not imperative for viral infectivity, it is the major escape mechanism utilized by Env to evade SF12 neutralization. Therefore, immunogen design for silent-face bNAbs requires the consideration of ontogeny, paratope binding dynamics, Env glycan occupancy, and topography to achieve native-like antigens [19,21,84].

### MPER

The MPER epitope is effectively shielded by complex glycans at positions N88 and N625 on the gp160 viral surface and represents a highly conserved transient region revealed following Env binding to CD4. [85,86] The native quaternary epitope remains poorly defined although several crystal structural models have been derived alone and in combination with lipid molecules. Several bNAbs (e.g., 10E8, 2F5, 4E10, DH511, DH517, VRC42, VRC46, VRC43.01, LN01, and PGZL1) targeting the C-terminal MPER domain have been isolated in PLHIV. [87] Most MPER bNAbs target linear epitopes that are transiently exposed during the pre-hairpin intermediate state or after forming the six-helix bundle during viral and target cell membrane fusion. [88–91] Interestingly, despite the uniqueness of this region, bNAbs targeting the MPER epitopes have characteristics similar to those targeting other conserved epitopes on Env. bNAbs elicited against the MPER epitopes contain long CDRH3 loops to bind the Env pre-fusion structures in the transitory pre-hairpin conformation. [89,92] This restricts the flexibility of the MPER fusion peptides and may disrupt the stabilization of the six-helix bundle to prevent viral entry into target cells. [93] Notably, the host immune response to the rapid transition from membrane hemifusion to complete fusion is typically inadequate to effectively thwart viral infection. [88,93] Steric hindrance from the N88/N625 glycans and gp120 may elicit non-broadly neutralizing antibodies. Therefore, for neutralization, MPER bNAbs must engage and incorporate the N88/N625 glycans and bind to the base of gp120 during the transitory phase of viral infection. [92,94] Results from the MABGEL-1 trial (ISRCTN64808733) demonstrated the durability of 4E10 and 2F5 bNAbs as valuable templates for vaccine development and provided a strong rationale for the evaluation of 10E8 bNAbs using modified bispecific and trispecific platforms to improve epitope binding. [10,95] Furthermore, liposomal MPER peptide immunogens targeting the 2F5 bNAb epitopes in the HVTN-133 clinical trial (NCT03934541) induced clade B heterologous neutralizing antibodies. This suggests that eliciting 2F5 bNAbs through vaccination is feasible although obstacles to immunogen delivery and MPER sequence diversity need to be addressed. [96] Interestingly, nanodisc assembly technologies are being utilized to develop novel MPER bNAb immunogens for HIV-1 vaccine development. [87] Nanoparticles are advantageous in immunogen design as they offer modalities for improved delivery of immunogens to germinal centers consequently inducing CD4^+^ T follicular helper cells and bypassing the glycan-induced non-neutralizing epitopes responsible for diverting B-cell affinity maturation [61]. Nanoparticles could in essence facilitate prolonged exposure of immunogens in the germinal center leading to higher affinity maturation during the development of bNAbs [97].

### Current dominating HIV-1 vaccine immunogen-generating strategies

The glycan shield exhibits distinct folding dynamics coupled with Env sequence variability which presents a formidable barrier to eliciting most bNAbs. Nevertheless, the identification of monoclonal bNAbs in PLHIV provides optimism for developing vaccines to elicit broadly neutralizing antibodies. [38,87] Reviews of vaccine designs that utilize Env *N*-linked glycans in epitope formation have validated the need to overcome the glycan heterogeneity and the low glycan immunogenicity by using alternative vaccine strategies. [22,38,97] Approaches to engage glycans that block access to conserved epitopes while preserving the salient contribution of glycans to the folding and stabilization of the Env trimer are areas of active research. [38] To experimentally elicit bNAbs, sequential vaccination with sets of in silico*-designed* immunogens targeting deciphered maturation pathways of B cell clone/s producing the target bNAb are used.

The germline targeting approach is a leading strategy in vaccine regimens to elicit bNAbs. Germline-targeting (GT) immunogens are designed to recapitulate the events in developing occurring during the development of long-lived memory B cells. [98,99] This involves using non-native trimers as GT priming immunogens to activate naïve B cell precursors or bind to the unmutated/inferred intermediates of mature bNAbs class lineages. [99] GT immunogen priming is essential to naïve B cell activation as only B cells producing antibody precursors predicted to develop into functional bNAbs must be stimulated at this stage. The priming step recruits B cell precursors into the germinal centers where the distinct characteristics associated with bNAbs are developed through somatic hypermutation and affinity maturation. [61,100] Successful priming largely depends on the frequency of bNAbs-produing B cell precursors within the entire human B cell repertoire. [100,101] This approach has been validated for the VRC01 class of bNAbs. Recent data from animal models and human clinical trials show that precursors to VRC01 bNAbs can be activated using appropriately designed germline B cells priming immunogens. [100–102] VRC01 class bNAbs are more favorably primed in part due to the relative abundance of precursors in the human B cell repertoire and their ability to engage the CD4 binding site epitope without the dominance of a long heavy chain complementary determining region 3 (CDRH3). [100,103] However, considerable improbable mutations are required to achieve neutralization breadth and potency. Therefore, GT immunogen designs rely on bioinformatics and computational approaches to predict and select precursor B cell clones that can develop into mature bNAbs-producing plasma cells [61,103,104].

Sequential vaccination with sets of prime-boost immunogens is required to drive the activated precursor B cells into the germinal centers (GCs). [102] Vaccination induces the proliferation of antigen-activated precursor B cells and interaction with CD4 T cells. The subsequent transfer into the center of B cell follicles leads to the formation of GCs. [102,105,106] This vital step in developing mature bNAbs allows for selecting improbable nucleotide substitutions through somatic mutations and infrequent activation-induced cytidine deaminase (AID) activity. [107,108] Also, mutations within the framework region are required for antibody binding region flexibility. However, framework region mutations are less tolerated than mutations in the complementary determining regions and can adversely affect B cell fitness in the germinal center. [104] This implies that several obstacles must be overcome within the germinal centers to drive the acquisition and accumulation of improbable mutations. Notably, the success of B cell responses in the germinal centers correlates with precursor frequency, B cell receptor affinity for booster immunogen, antigen avidity, follicular Th cell (T_FH_) support, antigen delivery, and adjuvants. [102,105] Thus, immunodominance and competition from more frequent non-neutralizing antibody-producing B cells may outcompete bNAbs B cell precursors within the GCs. [105,106] An approach to prevail over immunodominance using glycan masking of immunodominant non-neutralizing epitopes on antigens has informed iterative engineered Env outer domain GT immunogens. [106] Data from humanized mouse models show this approach is viable for the VRC01 class of bNAbs. [109]Interestingly, sequential boosting may have limitations in memory B cells GC reentry and occupancy which are critical for acquiring high-affinity for antibody recognition of antigens by somatic hypermutation. [102,110] Also, diversification of the T cell compartment through clonal expansion of the T_FH_ mediates antibody affinity selection and prevents the emergence of autoantibody B cells. This immune tolerance checkpoint disfavors the development of some bNAbs that exhibit autoreactivity. [105,111] Therefore, immunomodulation using immune tolerance inhibitors, adjuvants, and formulations may be required to succeed in bNAbs maturation [54].

Conceptually, native or native-like Env trimers with native glycosylation are preferred as boosting immunogens in the germ-line targeting approach. Boosting with heterologous native-like trimers should promote affinity maturation and generate neutralization against diverse wild-type Env epitopes. [99,104] Thus far, eliciting bNAbs that neutralize circulating wild-type HIV-1 viruses remains to be achieved. However, clinical trials using the GT immunogens have shown promise and validated this approach [98].

Alternative strategies for eliciting bNAbs have been proposed to complement the GT approach. [61] In the B cell lineage design approach, immunogen templates bind with high affinity to unmutated, intermediate bNAbs ancestors or the transmitted/founder viral strain. [54,57,112] The priming immunogens are designed to recapitulate the earliest maturation pathways of mature bNAbs using clonal lineage analysis. The lineage-based design relies on the computational inference of heavy and light chain gene arrangements of bNAb clones isolated from elite controllers. [57,108,113] The paratopes of the identified unmutated and intermediate ancestors then serve as structural templates for designing recombinant monoclonal antibodies with high-affinity binding. [99,113] Theoretically, lineage-based boosting immunogens can target multiple intermediate memory B cell lineages to induce several neutralizing antibody clones with higher protective antibody titers. [57] However, the diversity of the human antibody repertoire limits this strategy. The clonal lineages of bNAbs identified in one elite controller may not be relevant to eliciting antibodies in the general population [113].

The mutation-guided approach is based on identifying functional improbable mutations acquired during the development of bNAbs. This approach aims to shorten the affinity maturation process upon immunization. [61,107] Mapping of B cell ontogeny suggests that improbable mutations are generally acquired during the early and intermediate phases of bNAbs development. Therefore, priming immunogens are designed to bind to inferred unmutated common ancestors (UCA) and select B cell receptors with the highest probability of acquiring improbable mutations. [114,115] The feasibility of this strategy in a prime-boost regimen has been demonstrated for the DH270 B cell clones targeting the V3 glycan epitope in mouse models. Interestingly, although intermediate DH270 UCA lineages were successfully induced, no accumulation of improbable mutations was observed. Also, better vaccine-induced antibody neutralization was elicited for improbable mutations altering binding contacts to glycans N332 and N301 [115].

The epitope-focused vaccine design aims to develop epitope scaffolds that mimic conserved regions on the Env glycoprotein. This approach requires delineating the Env-bNAbs complexes and the atomic structure of conserved epitopes targeted by neutralizing antibodies. [57,61] Epitope scaffolds should exhibit high affinity binding to target bNABs and after immunization, it should elicit generation of antibodies binding native or native-like antigens. The uniqueness of this approach is that different classes of bNAbs and not a single lineage can assess the antigenicity of epitope scaffolds. Inference for immunogen designs is derived from identifying and isolating bNAbs recognizing overlapping conserved epitopes. Epitope-focused immunogens targeting the CD4 binding site, fusion peptide, and the V3 glycan site have been designed. [57,116] Thus far, the ideal prime-boost immunogens capable of inducing a polyclonal response remain to be achieved. Notably, glycan shielding, conformational masking, and the diversity of the HIV-1 viral immune evasion mechanisms that conceal the sites of vulnerability are key bottlenecks for this strategy [57].

The current strategies to elicit bNAbs can be broadly categorized into germline targeting and antibody-guided structural-based approaches. The priming immunogens designed by identification of germline or unmutated common ancestors (UCA) of naïve B cells should activate and expand rare precursors. Studies in humans and animal models have validated this initial step. However, boosting immunogens capable of shepherding the activated B cell receptors through affinity maturation towards bNAbs remains to be achieved in humans. The boosting phase immunogens should ideally select B cell clones bearing functional improbable heavy and light chain mutations corresponding to those identified in native bNAbs. [61,98,99,116] The strategies summarized here have provided proof of concept that eliciting bNAbs is possible by developing appropriately designed immunogens (Table 1). However, as mentioned above, several obstacles remain in the search for an HIV-1 vaccine immunogen. These partly include the rarity of bNAb precursors in B-cell repertoire, coupled with distinct germinal center affinity maturation dynamics, self-tolerance and clonal deletion, Env epitope immunodominance, and glycan heterogeneity. Therefore, novel strategies for the HIV-1 immunogen development armamentarium are urgently required to overcome these obstacles.

**Table 1:** Summary of strategies used to generate vaccine protocols for bNAb generation

bNAbs generation strategy	Immunogen description	Note	References
B cell linage vaccine design	Immunogen templates are derived from well-established bNAb lineages (inferred or unmutated common ancestor) isolated from elite-controllers	This is the initial step of antigen selection in the germline targeting strategy	54, 57, 61, 99, 108, 112, 113
Germline targeting	This Vaccine strategy uses immunogens capable of high affinity binding to naïve B-cell precursors (UCA) of target bNAbs. Recombinant Env SOSIP trimers are modified in amino acid positions critical for high affinity binding to germline B cell receptor binding. The derived SOSIP trimers are in an open configuration that mimics native Env with mainly complex glycans attached. Sequential immunizations are designed to shepherd precursor cells into bNAbs	A germline-targeting immunogen may be optimized using structure-based and/or mutation-guided immunogen design. Examples include eOD-GT8 and eOD-GT8-glycan-deleted derivatives	^54,57,61,99–113^
Mutation-guided immunogen design	Immunogens are designed to induce essential improbable mutations that are critical for neutralizing activity acquired during bNAb affinity maturation. The immunogens are designed to select for these mutations and bind with high affinity to the selected bNAb lineage genes	This has been difficult to achieve in most reported immunization protocols	^61,107,114,115^
Epitope-focused vaccine design	Immunogens are developed to mimic neutralizing epitopes derived from structural analysis of bNAb-Env complexes	This can be used as a static immunogen or as a booster immunogen in germline targeting	^57,61,116^

### Non-cognate Env epitope strategy

Generally, native-like (cognate) Env trimers, and gp120 or gp41 monomers, elicit sporadic and modest antibody responses with poor or no CD4-binding site neutralization. Recently, we developed a novel strategy without structural modifications of cognate Env glycoproteins consisting of mutation-guided glycan shield manipulation, germline-targeting Env design, and epitope-focused immunogen design by focusing on Env unrelated protein (non-cognate) epitopes specifically recognized by broadly neutralizing monoclonal antibodies (bNmAb). This approach eliminates the host immune responses to non-neutralizing Env epitopes of either glycan or glycoprotein nature and focuses the antibody responses to neutralization-sensitive Env regions. Our alternative Env-modifying strategy is based on the generation of Env sequence-unrelated protein immunogens as replicas of select bNmAb paratopes [117–119].

**Fig. 2:**
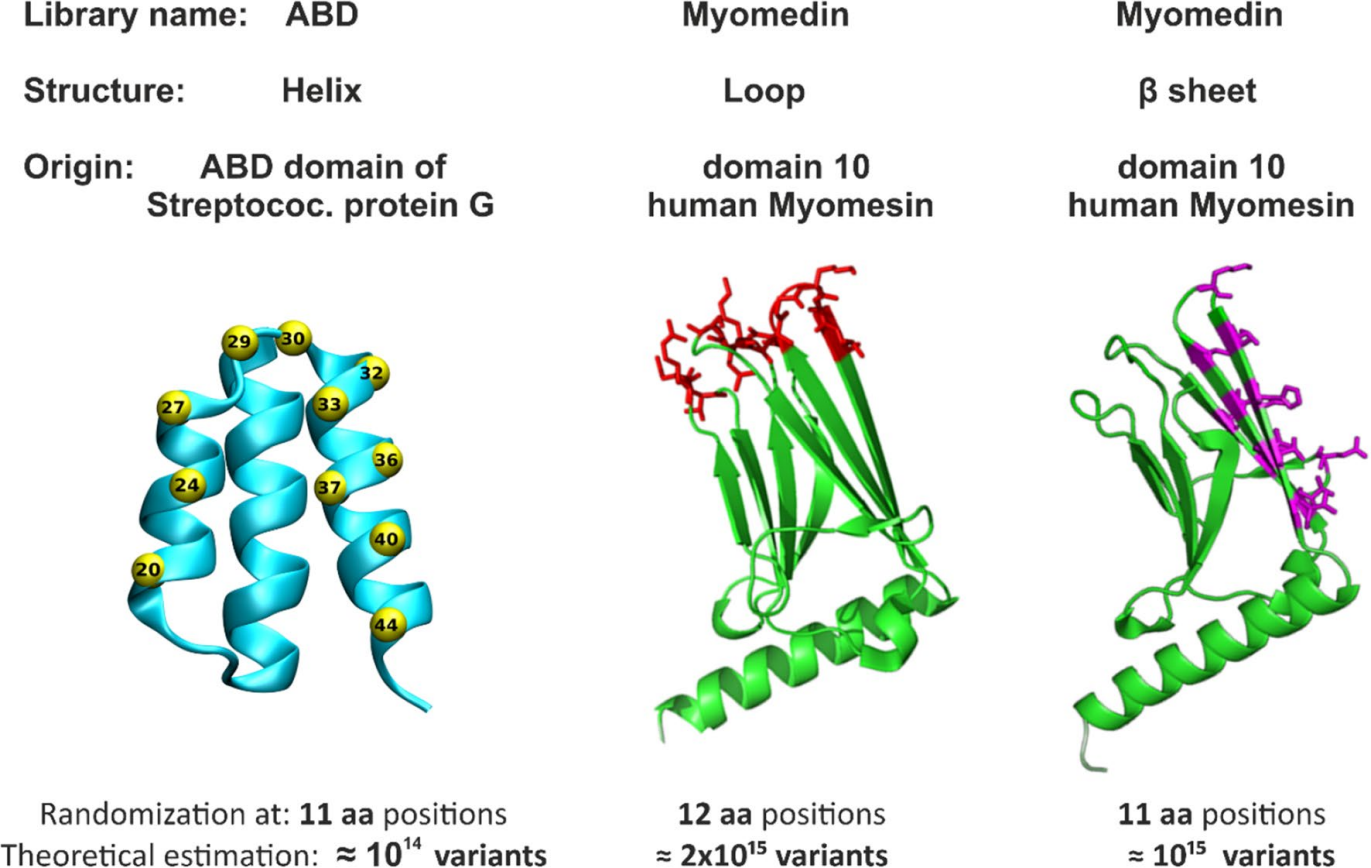
Development of highly complex combinatorial libraries based on protein domain scaffolds. Randomization of 11 amino-acid residues in two helices of the albumin-binding domain (ABD) in streptococcal protein G is shown in yellow. VRC01 bNAb was targeted by binders (VRA) from an ABD combinatory library with a 10^14^ theoretical complexity. Randomization of 12 amino-acid residues in domain 10 of the human muscle contractile protein Myomesin-1 loops L1-L3 generated a combinatorial library with a 2 × 10^15^ theoretical complexity. The Myomedin β sheet randomization generated a combinatorial library with a 10^15^ theoretical complexity. MPER bNAbs 10E8, PGT121, and PGT126 were targeted by binders MLA, MLB, and MLD respectively

This strategy was inspired by observations of anti-idiotype and anti-anti-idiotype antibodies generated during HIV-1 infection and was designated “Non-Cognate Ligand Strategy (NCLS)” based on comments by Klasse [120]. Briefly, panels of non-cognate ligands of bNmAbs were generated by screening small protein libraries developed by computer-assisted directed evolution of small binding proteins and mutagenesis of amino acids at discrete positions that were derived from two geometrically different protein domain scaffolds. First, we used a highly complex combinatorial protein scaffold library, generated by randomization of 11 discrete amino acid residues located in the second and third helices of the three-helix bundle albumin-binding domain (ABD) of streptococcal protein G (Fig. 2), which provided a theoretical complexity of 2 × 10exp14 variants. [121] A small 5 kDa protein scaffold was sufficient to mimic the epitope recognized by VRC01 bNAb, thus providing a collection of binders designated as **VRA mimotopes.** [118] To mimic larger and more complex Env epitopes involving *N*-glycans, we generated a second highly complex combinatorial library by randomization of 12 amino acid residues formed by 111-amino-acids in “domain 10” of human muscle contractile protein Myomesin-1 (designated Myomedin) (Fig. 2). We randomized residues of three Myomedin loops L1, L2, and L3, providing a “Myomedin loop combinatorial library” with an estimated complexity of 2 × 10exp15 variants. This Myomedin library was used to identify a set of binders mimicking the MPER epitope recognized by 10E8 bNAb and the immunogens were designated **MLA.** We also used the Myomedin library to mimic the glycan V3 loop Env epitopes recognized by PGT121 and PGT126 bNAbs and selected a set of binders designated **MLB** and **MLD** respectively that were used as immunogens. [117,119] The VRA, MLA, MLB, and MLD binders were individually tested as soluble immunogens in prime-boost vaccination protocols using experimental BALB/c mice. The hyperimmune sera from mice immunized with VRA, MLB, and MLD immunogens exhibited specific binding to recombinant multimerized HIV-1 gp120 protein. [27,118,119,122] Importantly, the neutralization potency against HIV-1 clades was assessed using panels of A, B, and C Tier 2 pseudoviruses, and the sera neutralized a substantial proportion of tested pseudovirus strains.

VRA-immunized mice produced Env-binding antibodies which neutralized up to 66% of the tested HIV-1 Tier 2 pseudoviruses. MLA immunizations elicited the production of Env binding antibodies which neutralized 54% of the tested pseudo viruses across clades A, B, C, and AE. MLD and MLB immunizations elicited murine serum antibodies that neutralized up to 40% and 45% respectively of the tested HIV-1-pseudotyped viruses across clades A, B, and C. Notably, all generated VRA, MLA, MLB, and MLD binders were produced as recombinant proteins in *E. coli* and thus their protein structure is not modified by attaching *N*- or *O*-glycans. For MLB and MLD we demonstrated that glycan-free protein immunogens can be generated to serve as effective immunogens mimicking Env V3 glycan-containing epitopes recognized by bNAbs PGT121 and PGT126.

As expected, sera from MLD-immunized mice neutralized eight pseudoviruses with the wild-type N332 glycan. Of five additional pseudoviruses that carried the N332 mutation and compensation by S334N PNGS, four of them were neutralized. [119] Conversely, two pseudoviruses lacking the N332 compensation mutation were not neutralized.

For the MLB and MLD immunogens, our observed virus-neutralizing coverage can be compared to that published by Walker et al. for PGT121 and PGT126 bNAbs. [51] MLB and MLD hyperimmune sera neutralized up to 45% and 40% respectively of similar pseudoviruses panels. [119] Also, due to the autoreactive properties of some bNAbs like 10E8, MLA-induced sera were tested for autoreactivity with murine cell antigens and were non-reactive [117].

Our preliminary immunization experiments with these binders formulated into corpuscular vaccines indicated that the multivalent formulation substantially enhances the Env-specific antibody titers and the hyperimmune sera neutralization coverage of PGT121 and PGT126 bNmAb. Also, a formulation of the generated binders as mRNA vaccines encapsulated in lipid nanoparticles could substantially enhance the titers of elicited neutralizing antibodies. [61] Further experiments will address questions regarding the use of these binders as priming antigens instead of germline-targeting precursors followed by sequential boosting with soluble multimeric Env constructs such as trimeric gp120, SOSIP variants, or membrane-bound recombinant Env formulations using nanodiscs. Therefore VRA, MLA, MLB, and MLD binders provide promising templates to develop HIV vaccine immunogens that can accommodate signature glycans and overcome the immune tolerance of the ENV trimer. Additionally, immunization protocols utilizing these binders within mosaic vaccine strategies could provide clues to countering escape variants that have influenced the vaccine efficacy of experimental HIV-1 vaccines.

## Conclusion and outlook

Thus far, glycan-dependent broadly neutralizing antibodies identified in elite viremic controllers are excellent templates to engage and manipulate the glycan shield. This review highlights the need for multipronged approaches that directly alter the immunodominant decoy glycans while focusing the broadly neutralizing antibody immune responses to the conserved epitopes on the Env glycoprotein. Moreover, to make the HIV Env glycan immunogenic through vaccination novel immunogens that utilize glycan immuno-shifting and focusing will be required. [38,123] We elaborated on the versatility of the Env glycan shield and the interactions with glycan-dependent broadly neutralizing antibodies. This review demonstrates the need to develop novel immunogen designs that solve glycan heterogeneity. The non-cognate ligand strategy using epitope-mimicking immunogens that utilize screening of highly complex small protein libraries has been effective in vitro. Therefore, in addition to the strategies targeting monoclonal bNAb-producing cell precursors, novel approaches based on the identification and determination of the molecular structures acting as non-cognate ligands of monoclonal bNAbs paratopes are promising alternatives.

Clinical-stage experimental vaccines routinely utilize non-glycosylated trimers in sequential immunization regimens. Although a native-like trimer with wild-type glycosylation patterns remains difficult to achieve, non-native trimers have advanced the field of HIV vaccine and cure research. Non-native like trimers often display glycosylation patterns analogous to monomeric gp120 in an open conformational state with reduced glycan occupancy at the potential N-linked glycosylation sites. [124] Thus, trimers presented in stabilized open conformational states induce non-neutralizing antibody responses. Conversely, a pre-fusion native-like trimer should in theory be able to induce broadly neutralizing antibody responses. [4,124] Overcoming the glycan barriers to eliciting bNAbs requires glycan profiling and glycosylation prediction of the Env glycoprotein conserved epitopes and the native bNAb-viral complexes. [41] Antigens with native-like structures, folding, and binding characteristics identified using glycosylation predictive tools will provide opportunities to accelerate the development of an HIV vaccine [41,125].

## Data Availability

The datasets used during the review are available from the corresponding author upon reasonable request.

## Abbreviations

ABD: Albumin binding domain
ADCC: Antibody-dependent cellular cytotoxicity
ADCD: Antibody-dependent complement deposition
ADCVI: Antibody-dependent virus inhibition
ART: Antiretroviral therapy
bNAbs: Broadly neutralizing antibodies
bNmAb: Broadly neutralizing monoclonal antibodies
CDRH: Complementary determining region heavy chain
CDRL: Complementary determining region light chain
Env: Envelope glycoprotein
HIV: Human immunodeficiency virus
HLA: Human leukocyte antigen
MPER: Membrane proximal external region
NCLS: Non-cognate ligand strategy
nNAbs: Non-neutralizing antibodies
PLHIV: People living with HIV
PNGS: Potential *N*-linked glycosylation sites
SHM: Somatic hypermutations
UCA: Unmutated common ancestor
